# To err is human; acute appendicitis a mistaken clinical identity for metastatic follicular thyroid carcinoma, a case report

**DOI:** 10.1016/j.ijscr.2022.107792

**Published:** 2022-11-22

**Authors:** Ahmed Jusabani, Neelam Ismail, Zainab Fidaali, Ramadhani Mjejwa, Tausi Maftah, Allyzain Ismail

**Affiliations:** a Department of Radiology, The Aga Khan Hospital, Dar-es-Salaam, Tanzania; b Department of Family Medicine, the Aga Khan Hospital, Dar-es-Salaam, Tanzania; c Department of Nuclear Medicine, Ocean Road Cancer Institute, Tanzania; d Department of General Surgery, The Aga Khan University, East Africa Medical College, Tanzania

**Keywords:** Follicular thyroid carcinoma, Acute abdomen, Interventional radiology, Case report

## Abstract

**Introduction and importance:**

Thyroid cancer accounts for majority of endocrine cancers with follicular thyroid cancer the second most common. It tends to spread via hematogenous route with distant metastasis thus besides presenting as a neck mass it may also present with symptoms tallying to regions of metastasis which may misguide the diagnosis. We report a case of a 50-year-old man who presented with features of appendicitis only to discover metastatic right iliac bone follicular thyroid cancer. We describe our experience on diagnostic formulation, radiological work up, surgery, radioactive iodine therapy and follow up.

**Case presentation:**

50-year-old man presenting with acute abdomen and fevers with suspicion for appendicitis, worked up and found to have metastatic follicular carcinoma. Underwent total thyroidectomy and radioactive iodine therapy to achieve disease stability without progression with a 5 year follow up completed.

**Clinical discussion:**

The tendency to jump to medical imaging to establish a diagnosis in a lab oriented rather than clinical oriented approach. The role of radiology to establish the underlying disease and identify the primary lesion. Successfully halting disease progression for metastatic follicular thyroid carcinoma with surgery and radioactive iodine therapy.

**Conclusion:**

Right iliac fossa tenderness does not always equate to acute appendicitis hence the use of diagnostic imaging to diagnose the metastatic lesion thus simplifying the puzzle to identify the primary. We hope through sharing our experience, we encourage the use of interventional radiology in a region that tends to opt for open approach when percutaneous approaches have shown to be successful.

## Highlights

• Metastatic follicular thyroid carcinoma mimicking acute appendicitis
• Use of intervention radiology to diagnose the underlying condition
• Successful disease halt of metastatic follicular thyroid carcinoma
• Serial imaging to assess disease progression

## 1 Introduction and importance

Thyroid cancer accounts for majority of endocrine cancers with papillary and follicular considered well differentiated cancers [1]. Follicular thyroid carcinoma (FTC) is the second most common thyroid cancer after papillary and although risk factors and management approach are similar among the two, important clinical and diagnostic differences do exist [2], [3]. Lymph node involvement is quite common in papillary whereas about only 10 % of follicular neoplasms spread to lymph nodes [4]. Instead, FTC has a tendency to spread via a hematogenous route with distant metastasis at presentation in up to 15 % with common sites of metastasis being bone and lung whereas brain, liver, and skin are less common sites [5], [6]. Therefore, other than presenting as a neck mass it may also present with symptoms tallying to regions of metastatic involvement such as back pain, cord compression, pathological fractures, difficulty breathing and abdominal pain which may misguide the provisional diagnosis.

Treatment for FTC is similar to papillary cancer despite numerous biological differences hence treatment strategies have been based off evidence from papillary cancer data due to lack of randomised controlled trials for FTC [7]. These strategies depend on extent of local disease, degree of metastasis, functional status of the patient and resistance to radioactive iodine (RAI). This includes surgery, RAI administration, radiation, and as last resort chemotherapeutic agents [8].

We report a case of a 50-year-old man who presented with features of an acute abdomen and fevers suspected of appendicitis only to discover metastatic right iliac bone FTC on workup. We describe our experience on radiological diagnostic formulation, oncological work up, surgical resection, RAI therapy and follow up. This paper has been reported in line with the SCARE 2020 criteria [9]. This article has been registered with the Research Registry with identification number researchregistry8181 and can be found through the following hyperlink Browse the Registry - Research Registry.

## 2 Case presentation

A 50-year-old male referred to the surgical clinic due to suspicion for appendicitis from a periphery outpatient clinic presented with severe right lower abdominal pain and anorexia with subjective fevers. Had prior bouts of right lower abdominal pain for the last month and treated with over-the-counter analgesia however on this presentation pain was intolerable. No history of prior trauma, prior surgery, no known allergies nor known comorbid. During the course of this attack visited a periphery clinic and verbal report from an abdominal ultrasound showed features suspicious for appendicitis hence referred to our centre.

**Fig. 1:**
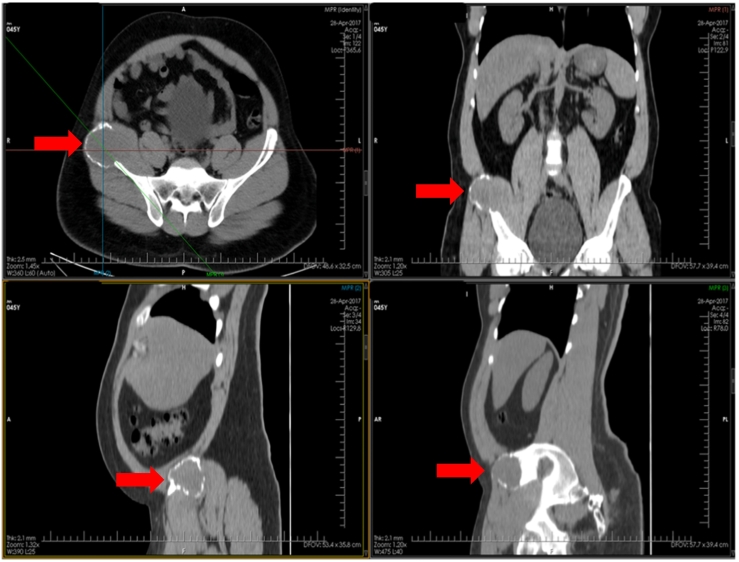
Initial CT scan of abdomen (axial, coronal and sagittal view) – Reformatted images of non contrasted CT abdomen showing an expansile hypodense mass involving the right iliac bone (red arrow).

On examination he was alert, oriented, not pale with stable vitals. Had significant right iliac fossa (RIF) tenderness and a positive psoas sign with rest of abdominal examination normal. Due to high suspicion for appendicitis an urgent computed tomography (CT) of the abdomen was performed which revealed a right iliac bone expansile lytic lesion with enhancing soft tissues abutting the iliacus muscle medially and gluteus minimus posterolateral suspicious for a metastatic bone lesion (Fig. 1).

**Fig. 2:**
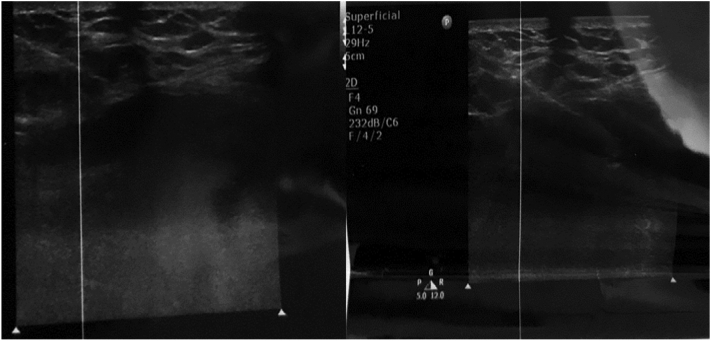
Images of ultrasound guided percutaneous biopsy of right iliac bone mass.

After initial resuscitation, pain control and baseline work up which revealed an elevated alkaline phosphatase in keeping with a lytic metastatic bone lesion, he was scheduled for an ultrasound guided biopsy by a consultant radiologist of the right iliac crest lytic lesion (Fig. 2). Histological findings of the lytic bone lesion revealed diffuse infiltration of malignant follicles that were lined by follicular cells and contained luminal colloid with scalloping of the colloid within the periphery of the follicles with immunohistochemistry staining for thyroid transcription factor-1 suggestive of a metastatic FTC. A thorough examination post biopsy results revealed a nodular right anterior neck mass which moved on swallowing and presence of mild tracheal shift hence referral to oncology for adequate work up.

**Fig. 3:**
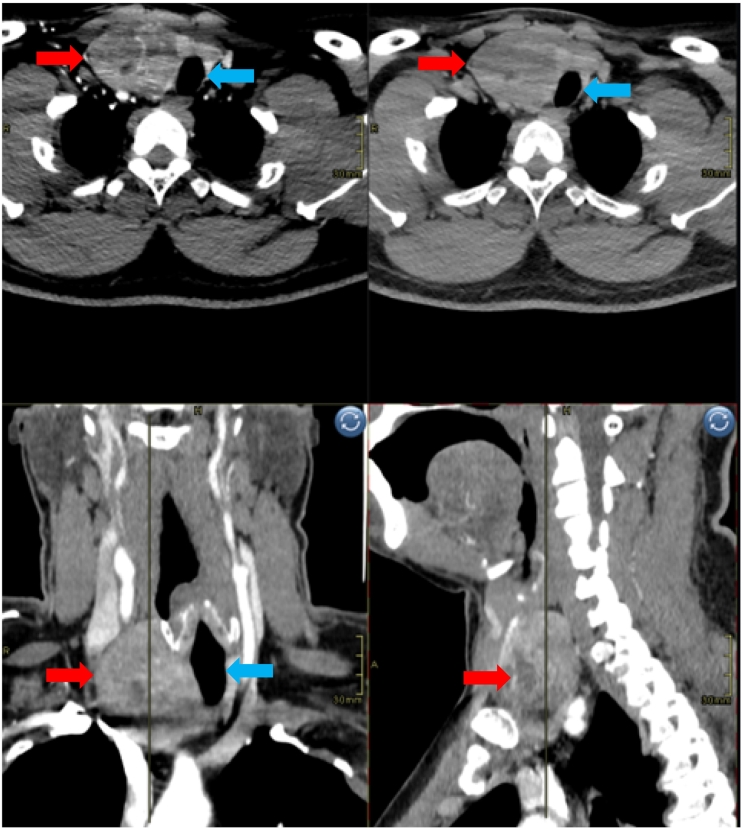
CT scan of neck (axial, coronal and saggital view) – Reformatted images of CT neck pre and post contrast showing a heterogneously enhancing solid mass involving the right thyroid lobe (red arrow) compressing and displacing trachea to left (blue arrow).

**Fig. 4:**
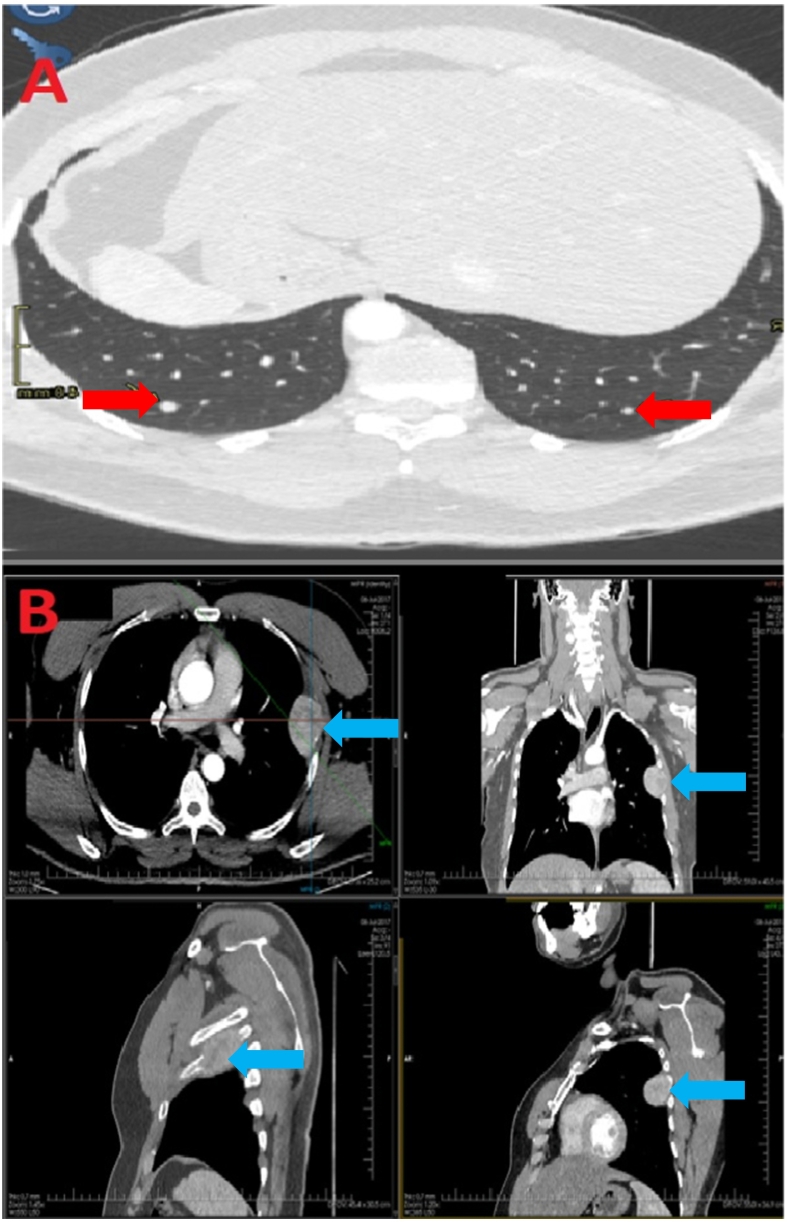
A - Metastatic work up axial view of CT chest lung window showing bilateral metastatic pulmonary nodules (red arrow). B – Metastatic work up reformatted CT chest images soft tissue window showing an enhancing lesion affecting the left 5th rib (blue arrow).

Oncological work up on CT neck revealed a heterogenous right thyroid mass with displacement of the trachea and multiple cervical lymph nodes suggestive of a primary thyroid malignancy (Fig. 3). Of note, multiple pulmonary nodular lesions in the lower zones and left 5th rib heterogenous mass suggestive of metastatic disease on CT chest (Fig. 4). A Tc-99m methylene diphosphonate whole body bone scan revealed findings consistent with a metastatic lesion in left scapula, right iliac bone, right ischium, multiple ribs and L4 vertebra. Abdominal imaging was otherwise normal, normal thyroid function tests but a significantly elevated thyroglobulin of above 30,000 ng/mL. A final diagnosis of metastatic FTC was made for tumour board discussion with radiologist, oncologist, nuclear physician, and general surgeon which concluded for the patient to undergo total thyroidectomy, followed by RAI.

**Fig. 5:**
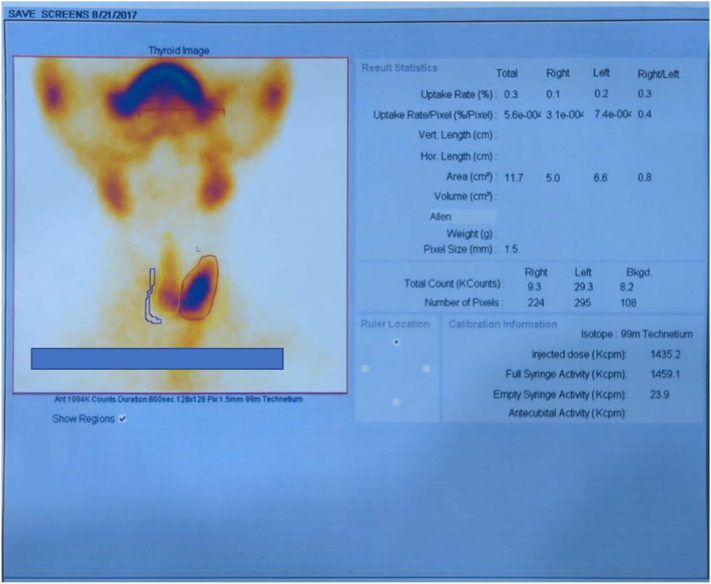
Radionuclide thyroid uptake scan post-operative revealing residual thyroid tissue.

**Fig. 6:**
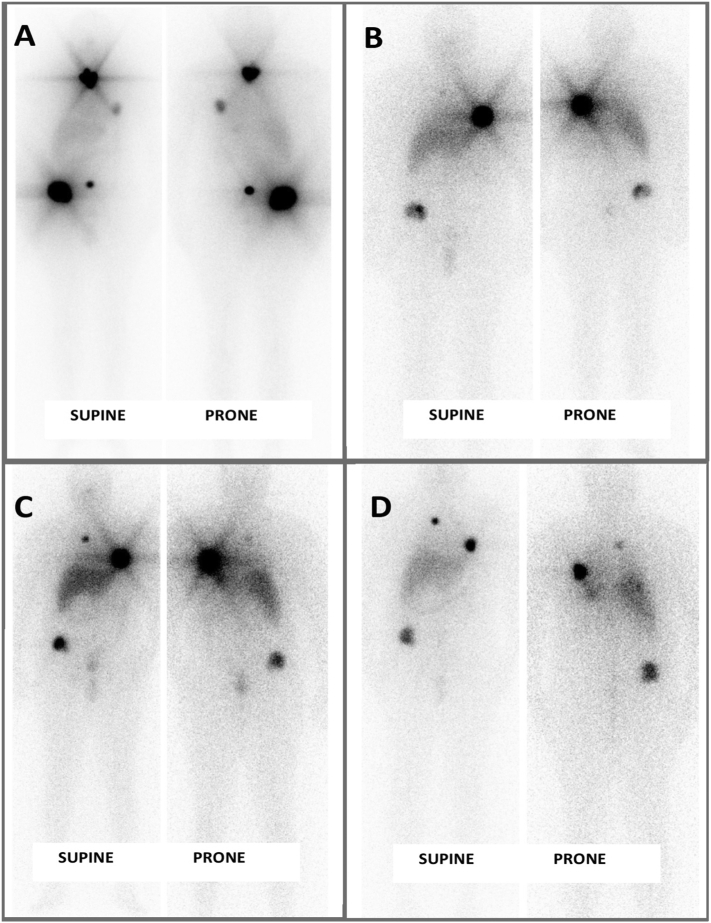
Serial iodine whole body scans at 6-month intervals after receiving RAI revealing response to treatment, stability of disease lesions without progression of disease (A – after 1st dose, B – after 2nd dose, C – after 3rd dose, D – after 4th dose).

Following total thyroidectomy histological analysis was consistent with a right lobe FTC and left lobe was found to have benign findings without tumour involvement. A postoperative radionuclide thyroid uptake scan (Fig. 5) to assess for remaining tissue revealed minimal residual thyroid tissue hence re-excision for completion of total thyroidectomy was done and achieved. Postoperative, he received RAI at dose of 200 mCi I-131 with 3 further subsequent doses at 6-month intervals to receive a total of 4 doses with serial post therapy whole body Iodine scans revealing a reduction in lesions with stable disease without evidence of disease progression (Fig. 6). In between RAI therapies, he was on levothyroxine to resolve hypothyroidism symptoms as well as to achieve thyroid stimulating hormone suppression.

Currently he is 5 years post-surgery with the most recent RAI received 2 years ago without significant complains and has returned to daily function and resumed occupation of a driver. He is currently asymptomatic with daily thyroxine supplements and biochemical thyroid hormones assessment every 3 months for thyroxine dose adjustment.

## 3 Discussion

Right iliac fossa pain immediately raises suspicion for appendicitis especially when coupled with features for infection and gastrointestinal symptoms. It creates diagnostic uncertainty with tendency towards a thought block for other differentials and to rule out appendicitis first due to the severity of complications for a delayed appendicitis diagnosis and treatment [10], [11]. A CT abdomen is the gold standard for diagnosing as it aids in conforming the diagnosis, a quick investigation as well as helps to look for other probable causes for RIF tenderness [12]. As with our case due to RIF tenderness coupled with subjective fevers the initial thought process was towards appendicitis with an urgent CT carried out only to be surprised with features of a metastatic iliac bone lesion. With the introduction of CT scans, it has now brought about a tendency for physicians to fish for a diagnosis guided by the chief complaint and brief medical history and examination [13]. This is seen due to the convenience of CT scans as well as the necessity of confirming diagnosis via a laboratory oriented rather than a clinical oriented approach towards patients [14].

The role of radiology has evolved from merely medical imaging for diagnostic purposes to utilisation of medical imaging approaches to aid in interventional diagnostic and therapeutic uses [15]. In sub-Saharan Africa there is a lack of interventional radiologists due to lack of resources and expertise hence the conventional open approach for even biopsies for histological analysis is still employed [16], [17]. Once a metastatic disease is identified the oncological puzzle to look for the primary begins [18]. Via the use of interventional ultrasound guided biopsy of the lytic bone lesion we could conclude on a metastatic FTC hence direct our approach towards thyroid cancer rather than blindly looking for a primary.

Follicular thyroid carcinoma has an exceptionally good prognosis for localised cancer however metastatic FTC with bone metastasis has a 5-year survival rate of around 60–70 % due to good response to surgery followed by RAI and/or external beam radiotherapy [19], [20]. It has a higher tendency to metastasize to bone compared to other forms of thyroid cancer with trends towards bone then lungs via a hematogenous route [6]. As seen with our case, he presented with metastatic lesions to both bone and lungs with his presenting feature of symptomatic right iliac pain. He responded to surgery and RAI with halt in progression of disease completing his fifth year of survival on supportive symptomatic care without decrease in quality of life. In our setting thyroid related oncological data is lacking however a study from the national cancer institute assessing all differentiated forms of thyroid cancer who underwent surgery and RAI revealed an overall survival of 82 % at 2 years. It is interesting to note of those assessed 62 % had metastasis at presentation with FTC more likely to metastasize then papillary with likelihood of metastasis at 60 % and 30 % respectively [21].

To our knowledge this is the only reported case report of a metastatic FTC mimicking an acute abdomen. Other reports on acute cord compression, pathological fractures and respiratory compromise have been reported on due to its metastatic nature with symptoms tallying to sites of metastasis [22], [23], [24]. We hope through our successful experience in use of minimally invasive ultrasound guided biopsy to help guide towards localising a primary to encourage the use of interventional radiology despite its relative prematurity in our region.

## 4 Conclusion

Follicular thyroid carcinoma is notorious for distant metastasis to bone and lung with presenting symptoms due to its effect on site of metastasis. RIF tenderness does not always equate to appendicitis and surprises in diagnosis still do exist hence the use of interventional radiology to diagnose the metastatic lesion thus the puzzle to identify the primary was significantly easier. We hope through our experience we encourage the use of interventional radiology in a region that tends to opt for open approach when percutaneous approaches have shown to be successful due to lack of resource and expertise.

## Abbreviations

CT: Computed tomography
FTC: Follicular thyroid carcinoma
RAI: Radioactive iodine
RIF: Right iliac fossa

## Patient's perspective

I was shocked when I found out my pain was due to a thyroid cancer and undergoing surgery and radioactive treatment was challenging. However, I am pleased to have returned to my daily activities with a disease that is currently under control while I continue to follow up for my disease.

## Provenance and peer review

Not commissioned, externally peer-reviewed.

## Ethical approval

Case study is exempt from ethical approval in my institution.

## Funding

This research did not receive any specific grant from funding agencies in the public, commercial, or not-for-profit sectors.

## Guarantor

Prof. Ahmed Jusabani.

## Research registration number

1. Name of the registry: RESEARCH REGISTRY
2. Unique identifying number or registration ID: researchregistry8181
3. Hyperlink to your specific registration (must be publicly accessible and will be checked):
  Browse the Registry - Research Registry.

## CRediT authorship contribution statement

A.J.: Study conception, production of initial manuscript, collection of data, proofreading
N.I.: Revision of the manuscript, proofreading
Z.F.: Revision of the manuscript, proofreading
R.M.: Revision of the manuscript, proofreading
T.M.: Production of initial manuscript, collection of data
A.I.: Study conception, production of initial manuscript, collection of data.

## Declaration of competing interest

None.
